# Fibrilação Atrial (Parte 1): Fisiopatologia, Fatores de Risco e Bases Terapêuticas

**DOI:** 10.36660/abc.20200485

**Published:** 2021-01-27

**Authors:** Fatima Dumas Cintra, Marcio Jansen de Oliveira Figueiredo

**Affiliations:** 1 Universidade Federal de São Paulo São PauloSP Brasil Universidade Federal de São Paulo, São Paulo, SP - Brasil; 2 Universidade Estadual de Campinas CampinasSP Brasil Universidade Estadual de Campinas, Campinas, SP – Brasil

**Keywords:** Fibrilação Atrial/fisiopatologia, Arritmias Cardíacas/fisiopatologia, Fatores de Risco, Obesidade, Sedentarismo, Terapia Combinada

## Abstract

A fibrilação atrial é a arritmia sustentada mais comum na prática clínica com predileção pelas faixas etárias mais avançadas. Com o envelhecimento populacional, as projeções para as próximas décadas são alarmantes. Além de sua importância epidemiológica, a fibrilação atrial é destacada por suas repercussões clínicas, incluindo fenômenos tromboembólicos, hospitalizações e maior taxa de mortalidade. Seu mecanismo fisiopatológico é complexo, envolvendo uma associação de fatores hemodinâmicos, estruturais, eletrofisiológicos e autonômicos. Desde os anos 1990, o estudo Framingham em análises multivariadas já demonstrou que, além da idade, a presença de hipertensão, diabetes, insuficiência cardíaca e doença valvar é preditor independente dessa normalidade do ritmo. Entretanto, recentemente, vários outros fatores de risco estão sendo implicados no aumento do número de casos de fibrilação atrial, tais como sedentarismo, obesidade, anormalidades do sono, tabagismo e uso excessivo de álcool. Além disso, as mudanças na qualidade de vida apontam para uma redução na recorrência de fibrilação atrial, tornando-se uma nova estratégia para o tratamento de excelência dessa arritmia cardíaca. A abordagem terapêutica envolve um amplo conhecimento do estado de saúde e hábitos do paciente, e compreende quatro pilares principais: mudança de hábitos de vida e tratamento rigoroso de fatores de risco; prevenção de eventos tromboembólicos; controle da frequência; e controle do ritmo. Pela dimensão de fatores envolvidos no cuidado ao paciente portador de fibrilação atrial, ações integradas com equipes multiprofissionais estão associadas aos melhores resultados clínicos.

## Introdução

A fibrilação atrial (FA) é caracterizada pela completa desorganização da atividade elétrica atrial e consequente perda da sístole atrial com padrão eletrocardiográfico característico e de fácil reconhecimento. Entretanto, o diagnóstico é desafiador, uma vez que muitos pacientes se apresentam assintomáticos ou com sintomas fugazes, dificultando o registro da arritmia. É a arritmia sustentada mais comum na prática clínica afetando 3% da população adulta, com predileção para faixas etárias mais avançadas.^1^ Com o envelhecimento populacional, as projeções para as próximas décadas são alarmantes. Estima-se que o número de pacientes portadores de FA com idade superior a 55 anos será mais que o dobro em 2060, o que consumirá grande quantidade de recursos dos cofres públicos.^2^ Além da importância epidemiológica, a FA é destacada pelas suas repercussões clínicas, incluindo os fenômenos tromboembólicos, com aumento, em média, de 4 vezes a chance de um acidente vascular cerebral (AVC), além de ser associada ao maior risco de mortalidade por todas as causas e outras importantes condições, como insuficiência cardíaca.^3 , 4^A incidência ajustada para idade e a prevalência de FA é menor nas mulheres em comparação com os homens; contudo, não acontece com a morbimortalidade. A FA está associada ao maior risco relativo para mortalidade por todas as causas, AVC, mortalidade cardiovascular, eventos cardíacos e insuficiência cardíaca no sexo feminino.^5^

Pacientes com essa anormalidade do ritmo também são mais vulneráveis a hospitalizações. Em uma recente metanálise incluindo 35 estudos e 311.314 pacientes, a incidência de admissão hospitalar foi de 43,7/100 pessoas ao ano. As doenças cardiovasculares representam as maiores causas de hospitalizações; no entanto, as causas não cardiovasculares também são frequentes nesse grupo de pacientes, como câncer e doenças pulmonares.^6^

Este artigo tem por objetivo revisar aspectos fisiopatológicos, fatores de risco e bases para o tratamento. As diretrizes para prevenção de eventos tromboembólicos e a ablação por cateter serão abordadas em outros manuscritos.

### Mecanismos fisiopatológicos

**Figura 1:**
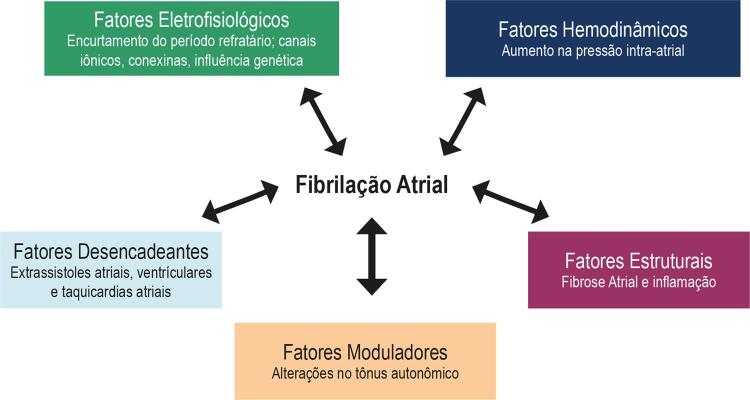
– Fatores fisiopatológicos implicados na gênese de fibrilação atrial.

Várias alterações fisiopatológicas levam à ocorrência de fibrilação, incluindo fatores hemodinâmicos, eletrofisiológicos, estruturais, autonômicos (moduladores), além de fatores desencadeantes representados pelas extrassístoles e taquicardias atriais ( Figura 1 ). Essas variam desde polimorfismos genéticos a modificações macroscópicas da estrutura do átrio, interferindo na atividade elétrica das células e resultando em desorganização da atividade elétrica atrial.

As propriedades elétricas do miocárdio são controladas por canais iônicos presentes na membrana celular. A ativação das células depende basicamente de canais de sódio, cálcio e potássio. O período refratário da célula depende, grosseiramente, do tempo entre a ativação da célula e o retorno do potencial de ação ao nível inicial. O aumento das correntes de influxo iônico (cálcio e sódio) prolonga o período refratário celular, e o aumento da corrente de efluxo (potássio) resulta no seu encurtamento. Outro componente importante na eletrofisiologia normal do coração são as conexinas, que são proteínas presentes nas junções entre os cardiomiócitos, responsáveis pela permeabilidade iônica entre as células, permitindo a propagação normal do impulso elétrico.^7^ Na FA, ocorrem alterações desses componentes da eletrofisiologia normal das células, o que se convencionou chamar de remodelamento elétrico. A sua forma mais comum decorre da entrada acentuada de cálcio nas células que se despolarizam com frequência aumentada. Esse aumento leva à inativação de correntes de cálcio e aumento de correntes de potássio, que resultam no encurtamento da duração do potencial de ação e aumento da vulnerabilidade à FA, além de favorecer a recorrência precoce após a cardioversão e a progressão de formas paroxísticas para formas mais persistentes da arritmia.^8^ Fatores genéticos podem estar relacionados a defeitos dos canais iônicos, e podem predispor à ocorrência de FA. Formas familiares da arritmia, embora sejam situações raras e heterogêneas, são bem descritas na literatura.^9 , 10^ O papel da genética na FA está sendo estudado, sendo uma corrente promissora na busca, cada vez mais atual, de formas de tratamento personalizado.

**Figura 2:**
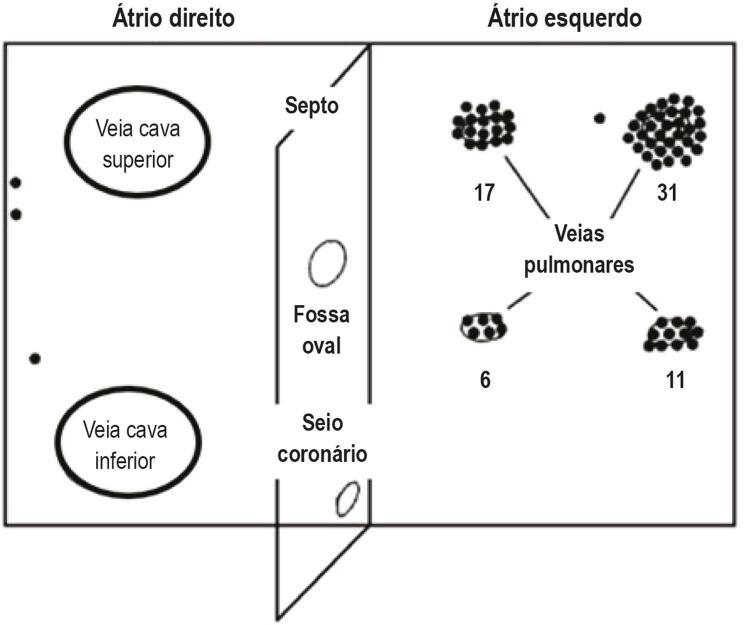
– Focos deflagradores de fibrilação atrial em vários pontos nos átrios (pontos escuros) provenientes, predominantemente, das veias pulmonares. Adaptada de Haïssaguerre M, Jaïs P, Shah DC et al.12 Spontaneous initiation of atrial fibrillation by ectopic beats originating in the pulmonary veins. N Engl J Med. 1998; 339(10):659–66.

Atualmente, as teorias mais aceitas para o início da arritmia e a sua manutenção são a presença de focos ectópicos como deflagradores da arritmia e a reentrada como fator de manutenção. Estudos iniciais já indicavam que a aplicação tópica de substâncias estimulantes no átrio, como a aconitina (alcaloide capaz de ocasionar bradicardia e hipotensão), no átrio originava taquicardia atrial rápida, que, por sua vez, induzia a FA.^11^ O estudo crucial no entendimento da origem focal da FA foi conduzido por Haïssaguerre et al.,^12^ no qual os autores, mapeando a atividade elétrica atrial em pacientes com FA, observaram focos ectópicos precoces que precediam a ocorrência da arritmia provenientes, principalmente, do interior das veias pulmonares ( Figura 2 ).

Enquanto a atividade focal é necessária para o início da FA, um substrato atrial propício para a sua manutenção é igualmente importante. Características estruturais, anatômicas e eletrofisiológicas são fundamentais para a ocorrência e a manutenção de circuitos de reentrada, que são tidos, atualmente, como fundamentais na manutenção da arritmia. A reentrada pode ser anatômica (com obstáculos criando zonas de condução lenta, como fibrose) ou funcional (refratariedade heterogênea, decorrente de propagação errática da frente de onda de ativação elétrica atrial). Essas condições aumentam a probabilidade de ocorrência de múltiplas ondas simultâneas de reentrada, facilitando a perpetuação da FA.^13^

A atividade autonômica também desempenha papel importante no início e na manutenção da FA.^14^ A ativação vagal pode alterar correntes de potássio dependentes da acetilcolina, com consequente redução da duração do potencial de ação podendo, assim, estabilizar circuitos de reentrada.^15^ Além disso, a ativação adrenérgica pode provocar o acúmulo de cálcio intracelular, o que pode deflagrar a arritmia.

Mudanças na estrutura do miocárdio atrial, particularmente a fibrose, separam as fibras musculares, interferindo na continuidade da condução do impulso elétrico, resultando em redução da velocidade de condução, que é fundamental para a reentrada.^16^ A fibrose leva à progressão da FA, podendo ser um alvo terapêutico^17^ e um preditor da resposta ao tratamento.^18^ Embora fatores eletrofisiológicos, incluindo o remodelamento elétrico, e morfológicos, como a fibrose e a dilatação atrial (remodelamento estrutural), sejam considerados os principais fatores envolvidos na fisiopatologia da FA, vem crescendo a quantidade de evidências que processos infecciosos ou inflamatórios podem permear e unir essas duas situações. Um estudo de caso controle com 56.870 participantes analisou a associação entre infecção pelo virus influenza, vacinação e risco de FA. Os autores demonstraram que a infeção aumentou o risco para o desenvolvimento da arritmia, ao passo que a vacinação apresentou efeito protetor em diferentes grupos de pacientes.^19^ A presença de infiltrado inflamatório, necrose celular e fibrose intersticial em pacientes com FA sem doença cardíaca estrutural documentada é maior em comparação com um grupo de pacientes sem a arritmia.^20^ Esses estudos vêm demonstrando maior concentração de mediadores ou de marcadores de atividade inflamatória, como a interleucina 6 ou a proteína C-reativa ultrassensível em pacientes com FA.^21^

### Fatores de risco para fibrilação atrial

O alto número de casos de FA encontrados na prática clínica não é apenas justificado pela idade – outros fatores contribuem para esse desfecho. Desde os anos 1990, o estudo Framingham em análises multivariadas já demonstrou que, além da idade, a presença de hipertensão, diabetes, insuficiência cardíaca e doença valvar é preditora independente dessa normalidade do ritmo.^22^ Entretanto, recentemente, vários outros fatores de risco são implicados, e as mudanças na qualidade de vida apontam para uma redução no número de casos de FA, tornando-se um novo pilar para o tratamento de excelência da FA.^23^

### Obesidade e fibrilação atrial

A obesidade, definida como índice de massa corporal (IMC) superior a 30 kg/m^2^, demonstra uma clara associação com a ocorrência de FA. Uma importante metanálise incluindo 51 estudos e 626.603 indivíduos demonstrou um aumento no risco de FA em 29% para cada aumento de 5 unidades no IMC. Além disso, o risco de FA pós-operatória e pós-ablação também foi 10% e 13% maior, respectivamente, para o mesmo aumento de peso.^24^ A progressão da doença da forma paroxística para a forma permanente também é mais significativa nos pacientes obesos conforme documentação em estudo coorte longitudinal com 21 anos de acompanhamento.^25^ A genética também parece justificar essa associação. Um estudo com mais de 50.000 indivíduos demonstra que as variantes genéticas associadas ao IMC alto se correlacionam com a incidência de FA, sugerindo uma relação causal entre as duas condições.^26^

A partir desse conhecimento, vários estudos prospectivos foram conduzidos para demonstrar o impacto da redução do peso na recorrência de FA.^27 - 32^ O estudo *Long-Term Effect of Goal-Directed Weight Management in an Atrial Fibrillation Cohort* (LEGACY) incluiu 355 pacientes, que foram acompanhados por 4 anos e divididos em três grupos de acordo com a perda de peso no final do estudo. Uma probabilidade 6 vezes maior de estarem livres de anormalidades do ritmo foi observada nos participantes com perda e manutenção de peso superior a 10% do peso corporal em comparação com o grupo que perdeu menos de 3% ou ganhou peso no período.^28^ Outro estudo, prospectivo e observacional, avaliou 149 pacientes com IMC superior a 27 kg/m^2^submetidos à ablação de FA e a um programa de redução de peso presencial, demonstrando maior tempo de sobrevida livre de eventos arrítmicos em comparação com o grupo controle.^27^ Resultados semelhantes foram observados em um estudo prospectivo com 4.021 pacientes obesos, em ritmo sinusal e sem história prévia de arritmia. O grupo foi submetido a cirurgia bariátrica ou tratamento conservador. A perda de peso observada no grupo com intervenção foi associada à redução significativa no risco de FA.^33^

Por outro lado, em uma análise secundária do estudo *Action for Health in Diabetes* (Look AHEAD) que avaliou pacientes com diabetes, a implementação de programa de perda de peso e atividade física não reduziu a ocorrência de FA.^34^ Um outro estudo populacional demonstra que a baixa massa magra também se relaciona com a presença de FA.^35^ Dessa forma, o real papel da distribuição de gordura corporal na arritmogênese ainda requer mais esclarecimentos, entretanto, é necessário reconhecer a obesidade como um fator de risco potencialmente modificável; e, em pacientes obesos e sobrepesos, a redução do peso em, pelo menos, 10% pode reduzir o risco de FA.

### Apneia obstrutiva do sono

Apneia obstrutiva do sono (AOS) é caracterizada pela obstrução completa ou parcial, recorrente das vias aérea superiores durante o sono, resultando em períodos de apneia, dessaturação de oxi-hemoglobina e despertares noturnos frequentes. O reconhecimento dessa anormalidade do sono por parte dos cardiologistas tornou-se fundamental após as publicações demonstrando aumento na mortalidade cardiovascular nos pacientes com AOS não tratada.^36^ Vários fatores contribuem para o dano cardiovascular nesses pacientes e, possivelmente inúmeros mecanismos estejam envolvidos. Entretanto, três fatores principais merecem destaque: hipóxia intermitente, despertares frequentes e alterações na pressão intratorácica. Essas alterações acabam por desencadear hiperatividade do sistema nervoso simpático; disfunção endotelial e inflamação.^37 - 40^ A ativação simpática observada nesses pacientes é um importante fator que, em parte, justifica a elevada prevalência de arritmias cardíacas nessa população, incluindo a FA. Além disso, a AOS pode prejudicar o funcionamento do átrio esquerdo. Estudos com ecocardiografia tridimensional demonstraram disfunção e remodelamento atrial esquerdo com reversão após tratamento efetivo com pressão positiva.^41 , 42^

Em um estudo epidemiológico, a ocorrência de arritmias cardíacas noturnas foi mais frequente nos pacientes portadores de AOS grave, definida como índice de apneia/hiponeia (IAH) superior a 30 eventos/hora. A FA ocorreu em 1,65% dos casos de AOS grave e 0,2% nos controles (p=0,03).^43^ Em outra análise de pacientes em acompanhamento ambulatorial por FA crônica em um hospital terciário submetidos à polissonografia basal, 81,6% apresentavam AOS.^44^ De fato, AOS e FA são condições que compartilham fatores de risco como idade, sexo, obesidade, hipertensão e insuficiência cardíaca e, dessa forma, a demonstração de casualidade é desafiadora na literatura científica.

Em um estudo prospectivo^45^ com pacientes referidos para a cardioversão elétrica de FA/ *flutter* atrial, observou-se 82% de recorrência nos pacientes com AOS sem tratamento ou com tratamento inadequado e 42% de recorrência nos pacientes tratados (p=0,013). Além disso, no grupo de pacientes não tratados, a recorrência foi ainda maior entre os que apresentavam maior queda na saturação de oxigênio durante o evento de apneia (p=0,034). O tratamento da AOS reduz o risco de recorrência de FA não somente em pacientes submetidos à cardioversão elétrica, mas também após ablação por cateter. Em estudo com 426 pacientes submetidos ao isolamento elétrico das veias pulmonares, 62 pacientes apresentaram AOS confirmada pela polissonografia, sendo 32 pacientes usuários de CPAP e 30 pacientes sem tratamento. O uso do CPAP foi associado a uma maior taxa de sobrevida livre de FA quando comparado ao grupo sem o uso do CPAP (71,9% *vs.* 36,7%; p=0,01). Os autores concluíram que o tratamento com CPAP em pacientes portadores de AOS submetidos a tratamento percutâneo da FA melhora a recorrência da arritmia, e nos casos de AOS sem tratamento adequado, o isolamento elétrico tem pouco valor terapêutico.^46^ Uma metanálise foi, então, realizada para determinar o papel da AOS no paciente portador de FA submetido à ablação por cateter, concluindo-se que a presença de AOS é associada ao maior risco de recorrência de FA após ablação (RR 1,25, 95% CI 1,08 a 1,45, p=0,003).^47^

Concluindo, a presença de AOS é alta em pacientes com FA, e os dados atuais sugerem a presença de uma relação dose-resposta entre a gravidade da AOS e a recorrência de FA. O tratamento adequado dessa anormalidade do sono reduz a recorrência clínica de FA mesmo em pacientes submetidos à ablação por cateter. Dessa forma, a adequada investigação e o tratamento, caso necessários, são uma medida importante no manejo clínico desses pacientes.

### Atividade física e fibrilação atrial

A inatividade física é um problema de saúde pública associado ao aumento das doenças cardiovasculares, insuficiência cardíaca, AVC, câncer, obesidade, diabetes tipo 2 e hipertensão.^48^ Sendo assim, propicia diversos fatores de risco para FA; contudo, mais recentemente, a literatura sugere a inatividade física como um fator de risco independente para FA. Cinco estudos populacionais demonstraram uma clara relação entre inatividade física e aumento do risco de FA.^49 - 53^ O estudo *Cardiorespiratory Fitness on Arrhythmia Recurrence in Obese Individuals With Atrial Fibrillation* (CARDIO-FIT) avaliou o impacto do ganho na capacidade cardiorrespiratória na ocorrência de FA em pacientes obesos e com sobrepeso.^32^ A cada equivalente metabólico adquirido durante o acompanhamento associou-se 9% de redução na recorrência da arritmia mesmo após a correção para o peso e fatores de risco. Um estudo com pacientes portadores de FA permanente submetidos a 12 semanas de exercício moderado a intenso foi relacionado a um aumento significativo da qualidade de vida quando comparado aos controles.^54^ Esses achados foram reprodutíveis por outros estudos randomizados e controlados, e a metanálise resultante demonstra que o treinamento físico melhora a capacidade ao esforço, qualidade de vida e fração de ejeção do ventrículo esquerdo.^55^

Por outro lado, a relação entre atividade física e FA parece não ser linear, e sim uma curva em “u”, ou seja, os extremos – sedentarismo ou a prática extenuante de exercícios – aumentam o risco de FA.^56^ Vale lembrar que se trata da prática de exercícios com doses muito altas que excedem a recomendação e correspondem a uma porcentagem muito pequena da população. De forma interessante, o efeito do exercício intenso parece ser influenciado pelo sexo. Uma metanálise sobre o assunto demostrou que a atividade física vigorosa foi associada a um aumento significativo no risco em homens (OR: 3,30; 95% CI: 1,97 a 4,63; p=0,0002); entretanto, a atividade intensa foi relacionada a uma redução ainda mais significativa no risco de FA em mulheres.^57^ Os mecanismos envolvidos nessa diferença de comportamento ainda não estão totalmente esclarecidos, mas o fato é que a prática de atividade física moderada deve ser encorajada como prevenção, tratamento e melhora da qualidade de vida em todos os pacientes portadores de FA.

### Outros potenciais fatores de risco modificáveis

Os efeitos do álcool no remodelamento atrial e no sistema nervoso autônomo podem, em parte, justificar a maior recorrência de FA nos indivíduos que utilizam álcool.^58^ Um estudo populacional com 109.230 participantes saudáveis e com consumo de álcool quantificado através de questionários demonstrou que, entre homens, o risco de FA aumentou de acordo com os quartis de uso semanal de álcool, sugerindo uma associação dose-resposta. O mesmo não foi verificado entre as mulheres.^59^ Mais interessante é a recente documentação de que a abstinência ao álcool se relaciona com a redução da recorrência de arritmia em pacientes com FA. Em um estudo multicêntrico, prospectivo e randomizado, realizado nos hospitais da Austrália, foram selecionados pacientes com consumo superior a 10 doses semanais e portadores de FA paroxística ou permanente, e que estavam em ritmo sinusal na avaliação basal. O grupo foi selecionado 1:1 para continuar com o uso habitual e a abstinência ao álcool. No total, 140 pacientes foram inclusos. A recorrência de FA ocorreu em 53% dos pacientes do grupo de abstinência e 73% no grupo controle. O tempo para a primeira recorrência foi maior no grupo de abstinência, e o numero total de eventos após 6 meses de acompanhamento foi significativamente menor nos que pararam com o uso em comparação aos controles.^60^

Os estudos que avaliaram a relação entre tabagismo e FA apresentaram resultados, inicialmente, discordantes; entretanto, uma metanálise incluindo 16 estudos prospectivos e 286.217 participantes demonstrou maior prevalência de FA entre os tabagistas, e a cessação do hábito foi associada à redução do risco.^61^ O tabagismo também influência negativamente os resultados do tratamento intervencionista da FA.^62^

Vale lembrar que o uso de altas doses de corticosteroides também foi relacionado ao aumento no risco de FA.^63^ Até o momento, não existem dados convincentes relacionando o uso de cafeína e o aumento do risco de FA; alguns estudos sugerem um modesto efeito protetor.^64^ O mesmo ocorre com transtornos de ansiedade. Em um recente estudo populacional com 37.402 adultos, não houve relação entre os sintomas de ansiedade ou depressão severa e FA.^65^

**Figura 3:**
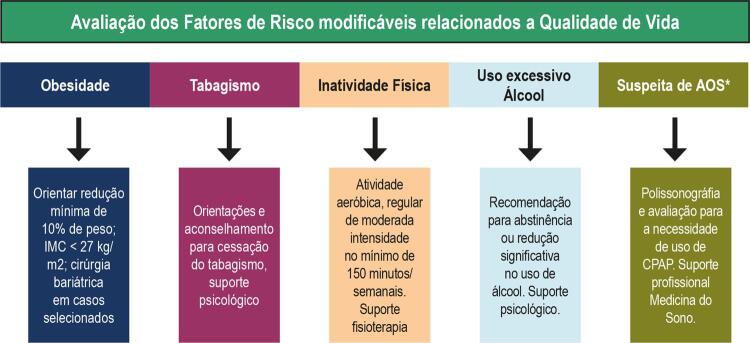
– Fatores de risco para fibrilação atrial relacionados à qualidade de vida e suas respectivas orientações.

A Figura 3 resume os principais fatores de risco modificáveis relacionados à qualidade de vida.

*Presença de ronco, sonolência excessiva diurna, fadiga, sono não reparador, alteração de memória

### Bases terapêuticas para fibrilação atrial

**Figura 4:**
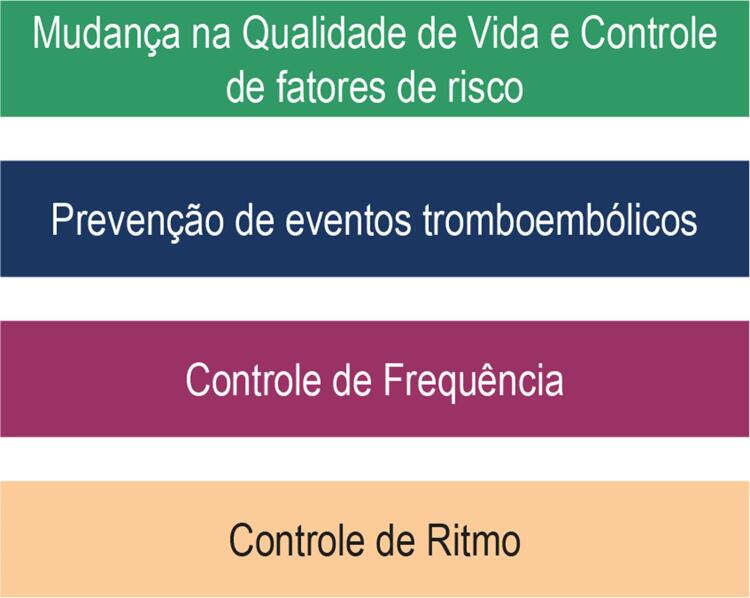
Pilares na abordagem terapêutica do paciente portador de fibrilação atrial

A abordagem terapêutica da FA envolve um amplo conhecimento do estado de saúde e hábitos do paciente e compreende quatro pilares principais: mudança de hábitos de vida e tratamento rigoroso de fatores de risco; prevenção de eventos tromboembólicos; controle da frequência; e controle do ritmo^66^ ( Figura 4 ). Serão mencionadas as bases terapêuticas relacionadas ao tratamento a longo prazo.

### Mudança na qualidade de vida e controle rigoroso de fatores de risco

Visa reduzir os fatores de risco modificáveis associados à qualidade de vida e ao tratamento rigoroso das comorbidades cardiovasculares. Dessa forma, além do controle do peso, tratamento do tabagismo, combate ao sedentarismo, uso comedido de álcool e otimização do padrão do sono, deve-se implementar o controle rigoroso de hipertensão arterial, diabetes e dislipidemia.

A hipertensão arterial é deletéria para o paciente com FA; além de constituir um fator de risco para eventos tromboembólicos, está associada a maior probabilidade de sangramento e recorrência dessa arritmia. Uma metanálise para prevenção de FA com o uso dos inibidores do sistema-renina angiotensina-aldosterona incluindo 87.048 pacientes provenientes de 23 estudos controlados e randomizados demonstrou que o uso desses medicamentos reduziu a probabilidade de ocorrência da arritmia em 33%, aproximadamente.^67^

Uma subanálise do estudo *Systolic Blood Pressure Intervention Trial* (SPRINT) avaliou as estratégias de controle intensivo da pressão arterial (PAS <120 mmHg) e controle convencional (PAS <140 mmHg) na ocorrência de FA. Após 5,2 anos de acompanhamento, foi observada uma redução de 26% no risco de FA no grupo de controle intensivo quando comparado com o controle convencional.^68^

Estudos demonstrando o benefício do controle da pressão arterial na redução do risco de FA foram reprodutíveis na literatura, incluindo pacientes com redução da fração de ejeção do ventrículo esquerdo,^69 , 70^ mas alguns dados contraditórios também estão disponíveis.^71 , 72^ Possivelmente, outros fatores devem influenciar a prevenção primária e secundária de FA no paciente hipertenso, e estudos ainda são necessários para o melhor esclarecimento dessa relação.

Em uma metanálise envolvendo 7 estudos de coorte prospectivos e 4 estudos caso-controle, incluindo 108.703 casos de FA, demonstrou-se que a presença de diabetes está associada ao aumento no risco de desenvolver essa arritmia em 34%, mesmo após ajuste para fatores de confusão.^73^ Os mecanismos fisiopatológicos dessa relação ainda estão em investigação, mas possivelmente são múltiplos, incluindo os impactos do diabetes no sistema nervoso autônomo como na neuropatia diabética. Além disso, a hiperglicemia, isoladamente, é capaz de aumentar o tônus simpático e diminuir o tônus parassimpático, o que pode facilitar a ocorrência da arritmia. O remodelamento elétrico e estrutural atrial associado ao estresse oxidativo também corrobora a FA. ^74^ Entretanto, a relação entre diabetes e FA torna-se mais importante após a documentação de que o controle rigoroso da glicemia está associado a um melhor controle da FA. Em uma análise com 12.605 pacientes, o tratamento do diabetes por 5 anos foi associado a uma redução de aproximadamente 30% nos casos de FA.^75^

O diabetes também pode prejudicar a evolução dos pacientes com FA submetidos à ablação por cateter. Em um recente estudo multicêntrico incluindo 7 centros de alto volume na Europa, foi demonstrada uma maior recorrência de FA em 1 ano no grupo diabético.^76^ O controle da glicemia também parece influenciar favoravelmente a evolução de pacientes submetidos à ablação. Uma análise observacional de pacientes após ablação demonstrou que o uso da pioglitazona foi associado a menor necessidade de um segundo procedimento ablativo.^77^

A relação entre dislipidemia e FA ainda está em investigação. Uma análise observacional incluindo dois grandes bancos de dados (MESA e Framingham) demonstrou que altos níveis de HDL estavam associados ao menor risco de FA, ao passo que altos níveis de triglicérides estavam associados ao seu maior risco. Nenhuma relação foi encontrada com o LDL.^78^ Por outro lado, em um estudo prospectivo populacional, não houve associação entre os níveis de HDL, triglicérides e FA, e baixos níveis de LDL foram associados ao maior risco de FA. Além disso, o uso de fármacos hipolipemiantes não influenciou a ocorrência de FA.^79^

Na verdade, essas análises pontuais voltadas a um único fator de risco falham em demonstrar ações combinadas que, normalmente, são aplicadas na prática clínica. Para avaliar esse efeito, foram selecionados 281 pacientes consecutivos submetidos à ablação por cateter com múltiplos fatores de risco, e foi oferecido um programa de controle agressivo desses fatores. Os pacientes submetidos ao programa apresentaram significativamente maior redução de peso e melhor controle da pressão arterial, glicemia e dislipidemia. Como consequência, apresentaram uma maior redução na frequência, duração e sintomas de FA quando comparado com o grupo controle (p <0,001).^80^

Prevenção de eventos tromboembólicos

A FA é uma arritmia em que a avaliação de elegibilidade para prevenção de eventos tromboembólicos é mandatória. O uso do anticoagulante pode prevenir a maioria desses eventos e prolongar a sobrevida. O anticoagulante é superior ao tratamento com ácido acetilsalicílico isolado ou associado ao clopidogrel. Deve ser instituído a todos os pacientes portadores de FA, exceto quando classificados com muito baixo risco ou na vigência de alguma contraindicação ao uso dessa classe de medicamentos.^81^ A oclusão do apêndice atrial esquerdo constitui uma segunda alternativa para prevenção de eventos tromboembólicos no paciente com limitações ao uso dos anticoagulantes.

### Controle da frequência cardíaca na fibrilação atrial

O controle da frequência cardíaca (FC) é parte integrante do tratamento do paciente com FA e, normalmente, é suficiente para reduzir os sintomas. O alvo terapêutico da FC ainda não está estabelecido na literatura. O estudo *Rate Control Efficacy in Permanent Atrial Fibrillation (* RACE) selecionou 614 paciente com FA permanente elegíveis ao controle da frequência randomizados para estratégia leniente (FC repouso <110 bpm) ou estratégia rigorosa (FC de repouso <80 bpm e inferior a 110 bpm no exercício moderado). O objetivo foi avaliar as duas estratégias em relação ao desfecho combinado de morte cardiovascular, hospitalização por insuficiência cardíaca, AVC, embolia sistêmica, sangramento e arritmias graves. Após um seguimento de 2 anos, não houve diferenças significativas entre ambas as abordagens, e a frequência de sintomas e eventos adversos foi semelhante entre os dois grupos.^82^ Em análise subsequente, a estratégia leniente também não foi associada a remodelamento cardíaco adverso.^83^

Os fármacos utilizados para essa finalidade incluem: betabloqueadores, bloqueadores de canais de cálcio (diltiazen, verapamil), digoxina ou combinação dessas substâncias.^84^ Vale lembrar que a amiodarona pode ser utilizada em casos selecionados.

Os betabloqueadores são considerados os fármacos de primeira linha para o controle da FC no paciente com FA pela sua boa tolerabilidade, redução dos sintomas e melhora funcional. As opções terapêuticas, as doses e os efeitos colaterais mais comuns estão demonstrados na Tabela 1 . Vale lembrar que, no insucesso terapêutico, a combinação de medicamentos pode ser tentada. No paciente com disfunção ventricular, o betabloqueador permanece como fármaco de primeira escolha pelos seus conhecidos benefícios nessa população, e a associação com digoxina pode ser tentada, quando necessário. Os bloqueadores de canais de cálcio não devem ser usados no paciente com insuficiência cardíaca com fração de ejeção reduzida pelo seu efeito inotrópico negativo.^84^ Por fim, a ablação do nó atrioventricular seguida de estimulação cardíaca artificial constitui uma opção terapêutica nos casos de falência da estratégica medicamentosa.

**Tabela 1:** – Medicamentos utilizados para o controle da frequência cardíaca em pacientes portadores de fibrilação atrial. Adaptada de ESC Scientific Document Group.84 2016 ESC Guidelines for the management of atrial fibrillation developed in collaboration with EACTS. Eur Heart J. 2016;37(38):2893-2962

Drogas mais utilizadas para o controle da Frequência cardiaca em pacientes com FA			
		Dose	Efeitos colaterais
Betabloqueadores	Metoprolol	100 a 200mg/dia	letargia, dor de cabeça, edema, sistomas respiratórios, alterações gastrointestinais, tontura, bradicardia, bloqueio atrioventricular, hipotensão
	Nebivolol	2,5 a 10mg/dia	
	Bisoprolol	1,25 a 20mg/dia	
	Carvedilol	3,125 a 50mg/ duas vezes ao dia	
Bloqueador canais de cácio	Diltiazen	60mg/ três vezes ao dia (máximo 360mg/dia)	tontura, mal-estar, letargia, dor de cabeça, edema, alterações gastrointestinais, bloqueio atrioventricular, hipotensão
	Verapamil	40 a 120mg/ três vezes ao dia (máximo 480mg/dia)	
Digoxina		0,0625 a0,25mg/dia	Alteração gstrointestinal, tontura, embaçamento visual, dor de cabeça, efeitos pro-arritmicos em doses tóxicas

### Controle do ritmo no paciente com fibrilação atrial

A reversão aguda ao ritmo sinusal e a terapia de manutenção do ritmo sinusal são importantes estratégias no manejo do paciente com FA. Apesar da manutenção do ritmo sinusal parecer, intuitivamente, superior quando comparada à estratégia de controle da frequência, não há forte documentação científica dessa afirmação. O estudo multicêntrico AFFIRM randomizou para essas duas estratégias de tratamento pacientes portadores de FA. Foram 4.060 pacientes com idade média de 69,7 anos, em que 70,8% apresentavam hipertensão arterial e 38,2%, doença da artéria coronária. Foram documentadas 310 mortes entre os pacientes alocados na estratégia de controle de frequência, e 356 nos pacientes com controle do ritmo após um seguimento médio de 3,5 anos com máximo de 6 anos (p=0,08). Além disso, o grupo submetido ao controle do ritmo apresentou mais efeitos adversos a medicações e um maior número de hospitalizações.^85^ Resultado semelhante foi observado no estudo RACE, em que o desfecho primário (morte e morbidade cardiovascular) ocorreu em 17,2% dos pacientes na estratégia controle da frequência e em 22,6% no controle do ritmo, após seguimento de 2,3 anos (p=0,11).^86^

Apesar de esses estudos não demonstrarem vantagem do controle do ritmo na sobrevida, alguns pontos devem ser mencionados. Uma subanálise do estudo AFFIRM utilizando modelos para determinar as relações entre sobrevida, variáveis clínicas basais e variáveis dependentes de tempo, demonstrou que a presença do ritmo sinusal e o uso de anticoagulante foram associados ao menor risco de morte. Por outro lado, o uso de fármacos antiarrítmicos foi associado ao aumento da mortalidade após ajuste para ritmo sinusal. Esses dados sugerem que o benefício do ritmo sinusal pode ter sido minimizado, e métodos alternativos para manutenção de ritmo sinusal com menores efeitos adversos podem ser promissores.^87^ Outra crítica a esses resultados é o tempo curto de seguimento. De fato, em uma análise populacional com seguimento superior a 5 anos, a mortalidade foi de 41,7% no grupo submetido à estratégia de controle do ritmo, e 46,3% no controle da frequência.^88^ Sendo assim, deve-se ter em mente que a escolha entre o controle do ritmo ou da frequência deve ser individualizada e, muitas vezes, é um processo dinâmico. Em um determinado momento, a estratégia de controle do ritmo pode ser atrativa, mas, em pacientes mais velhos com sintomas pouco expressivos, o controle da frequência pode constituir uma alternativa.

A reversão aguda ao ritmo sinusal é realizada por meio de cardioversão química ou elétrica, segundo os protocolos vigentes. Para a manutenção subsequente do ritmo sinusal, o uso de fármacos antiarrítmicos a longo prazo, a ablação por cateter ou a associação de estratégias são possibilidades que devem ser discutidas com o paciente. O uso de fármacos antiarrítmicos para manutenção do ritmo é comum no manejo clínico do paciente. A Tabela 2 demonstra os medicamentos utilizados para essa finalidade disponíveis no Brasil, com suas respectivas dosagens e efeitos colaterais. É importante mencionar que os efeitos colaterais dos fármacos antiarrítmicos usados a longo prazo são inúmeros, e foram expostos os mais comuns ou de maior gravidade. De fato, a escolha de tais medicamentos é estabelecida mais pelo seu perfil de segurança do que pela sua eficácia. O exemplo clássico é a amiodarona, que, apesar de apresentar superioridade frente a outros fármacos antiarrítmicos na manutenção do ritmo sinusal, tem seu uso restrito a pacientes com insuficiência cardíaca devido a seus efeitos tóxicos importantes com o uso prolongado.^81^ A propafenona e sotalol têm seu uso predominante no paciente sem doença cardíaca estrutural, lembrando que o sotalol pode prolongar o intervalo QT, e o monitoramento eletrocardiográfico é recomendado com o uso dessas medicações.

**Tabela 2:** – Fármacos antiarrítmicos utilizados para a manutenção do ritmo sinusal

Drogas utilizados para a manutenção do ritmo sinusal		
	Dose	Efeitos Colaterais
Propafenona	150 a 300mg / 3 vezes ao dia	Vertigem, palpitações disturbios da condução cardíaca, bradicardias, taquicardias, ansiedade, disturbios do sono, cefaéia
Sotalol	80 a 160mg / 2 vezes ao dia	Bradicardia, dispneia, dor no peito, palpitação, sincope, tontura, diarréia, nausea, vômito, fadiga, erupção cutânea, *torsade de pointes*
Amiodarona	100 a 200mg ao dia	neutropenia, agranulocitose, bradicardia, taquicardia. *torsade de pointes* , hipo e hipertireoidismo, neuropatia ótica, neurite, pancreatite, aumento de transaminases, transtorno hepático agudo, estado confusional, penumonite intersticial, brancoespasmo, eczema, urticária, hipotensão

A ablação por cateter visando ao isolamento elétrico das veias pulmonares é o tratamento intervencionista largamente utilizado para a prevenção de recorrência de FA. De modo geral, a ablação por cateter é superior aos fármacos antiarrítmicos na manutenção do ritmo sinusal^89^ e, atualmente, apresenta sua indicação em pacientes sintomáticos com FA paroxística ou persistente refratária ou intolerante a pelo menos um medicamento antiarrítmico, ou como primeira linha de tratamento na FA paroxística, sintomática de acordo com as preferências do paciente. Outras indicações individualizadas também podem ocorrer. O estudo CABANA comparou a ablação por cateter com a terapia medicamentosa otimizada em pacientes com FA paroxística e persistente de acordo com o desfecho combinado de mortalidade total, AVC, sangramento maior e parada cardíaca. Após 5 anos de seguimento, não houve diferenças significativas entre as duas estratégias,^90^ mas as análises relacionadas à qualidade de vida demonstram uma melhora clínica significativa, e também na qualidade de vida dos pacientes submetidos à ablação.^91^

### Cuidado integrado no cuidado do paciente com fibrilação atrial

Oferecer a complexidade de ações necessárias para o atendimento de excelência no paciente com FA é desafiador na prática clínica. Instituir mudanças na qualidade de vida, promover o controle rigoroso de fatores de risco, além da anticoagulação adequada e decisões relacionadas às diferentes estratégias terapêuticas quando centradas em um único profissional, podem ocasionar resultados insatisfatórios. Nesse sentido, a organização dos serviços de saúde com equipes multiprofissionais para o atendimento do paciente com FA é fundamental para assegurar o melhor atendimento. De fato, um estudo randomizado comparando o cuidado usual com o cuidado multidisciplinar demostrou uma redução no risco relativo de 35% no desfecho combinado de hospitalização e mortalidade.^92^ Outro ponto importante é que a ausência completa de eventos de FA, muitas vezes, é utópica, e o objetivo para o tratamento deve consistir em melhora da qualidade de vida, prevenção cardiovascular e mitigação das recorrências clínicas.
