# Supplementary material for: Fibrilação Atrial (Parte 1): Fisiopatologia, Fatores de Risco e Bases Terapêuticas
Source: Arq Bras Cardiol. 2021 Jan 27;116(1):129–39. [Article in Portuguese] doi: 10.36660/abc.20200485 (PMC8159512; doi:10.36660/abc.20200485)
Supplement: Translation

# Translation: Atrial Fibrillation (Part 1): Pathophysiology, Risk Factors, and Therapeutic Basis

**Authors:** Fatima Dumas Cintra, Marcio Jansen de Oliveira Figueiredo

**Affiliations:** 1 Brazil Universidade Federal de São Paulo, São Paulo, SP - Brazil; 2 Brazil Universidade Estadual de Campinas, Campinas, SP – Brazil

**Keywords:** Atrial Fibrillation/physiopathology, Arrhythmias Cardiac/physiopathology, Risk Factors, Obesity, Sedentarism, Combined Modality Therapy

## Abstract

Atrial fibrillation is the most common sustained arrhythmia in clinical practice, with a preference for older age groups. Considering population ageing, the projections for the next decades are alarming. In addition to its epidemiological importance, atrial fibrillation is evidenced by its clinical repercussions, including thromboembolic phenomena, hospitalizations, and a higher mortality rate. Its pathophysiological mechanism is complex and involves an association of hemodynamic, structural, electrophysiological, and autonomic factors. Since the 1990s, the Framingham study of multivariate analyses has demonstrated that hypertension, diabetes, heart failure, and valvular disease are independent predictors of this rhythm abnormality along with age. However, various other risk factors have been recently implicated in an increase of atrial fibrillation cases, such as sedentary behavior, obesity, sleep disorders, tobacco use, and excessive alcohol use. Moreover, changes in quality of life indicate a reduction in atrial fibrillation recurrence, thus representing a new strategy for excellence in the treatment of this cardiac arrhythmia. Therapeutic management involves a broad knowledge of the patient’s health state and habits, comprehending 4 main pillars: lifestyle changes and rigorous treatment of risk factors; prevention of thromboembolic events; rate control; and rhythm control. Due to the dimension of factors involved in the care of patients with atrial fibrillation, integrated actions performed by interprofessional teams are associated with the best clinical results.

## Introduction

Atrial fibrillation (AF) is characterized by the complete disorganization of atrial electric activity and consequent loss of atrial systole with a characteristic and easily recognizable electrocardiographic pattern. However, its diagnosis is challenging since many patients are asymptomatic or have fleeting symptoms, thus hindering its record. AF is the most common sustained arrhythmia in clinical practice, affecting 3% of the adult population and preferentially affecting older adults.^1^With population ageing, the projections for the next decades are alarming. The number of patients with AF aged over 55 years in 2060 is estimated to be more than twice that of 2010, which will demand enormous amounts of public resources.^2^In addition to its epidemiological importance, AF is evidenced by its clinical repercussions, including thromboembolic phenomena, increasing the chances of a stroke by 4 times; it is also associated with a higher risk of all-cause mortality and other important conditions such as heart failure.^3 , 4^

While the age-adjusted incidence and prevalence of AF is lower in women than in men, the same is not true for morbidity and mortality. AF is associated with a higher relative risk for all-cause mortality, stroke, mortality from cardiovascular causes, cardiac events, and heart failure in women.^5^

Patients with this rhythm abnormality are also more vulnerable to hospitalizations. A recent meta-analysis including 35 studies and 311 314 patients reported a hospitalization rate of 43.7/100 people per year. Cardiovascular diseases represented the biggest causes of hospitalization, but non-cardiovascular causes such as cancer and lung diseases were also frequent in this group of patients.^6^

This article aims to review pathophysiological aspects, risk factors, and basis for treatment of AF. Guidelines for preventing thromboembolic events and performing catheter ablation will be addressed in other manuscripts.

### Pathophysiological Mechanisms

Various pathophysiological alterations lead to fibrillation, including hemodynamic, electrophysiological, structural, and autonomic (modulatory) factors, as well as triggering factors represented by extrasystoles and atrial tachycardias ( Figure 1 ). These vary from genetic polymorphisms to macroscopic changes in atrial structure, interfering with the electrical activity of cells and resulting in disorganized atrial electrical activity.

**Figure 1:** – Pathophysiological factors implicated in the genesis of atrial fibrillation.

The electrical properties of the myocardium are controlled by ionic channels present on the cell membrane. Cell activation relies basically on sodium, calcium, and potassium channels. The cells’ refractory period roughly depends on the time between cell activation and the return of the action potential to its initial level. An increase in ionic influx (calcium and sodium) prolongs the refractory period, while an increase in potassium efflux results in a shortening of this period. Another important component of the normal electrophysiology of the heart are connexins: These are proteins present in the junctions between cardiomyocytes which are responsible for the ionic permeability between cells, allowing normal propagation of the electrical impulse.^7^In AF, there are alterations in these components of normal cell electrophysiology and these are named electrical remodeling. The most common form of electrical remodeling results from an acute entry of calcium into the cells, which depolarize with an increased frequency. This leads to the inactivation of calcium currents and to an increase in potassium currents, resulting in a shortened duration of the action potential and in increased vulnerability to AF, in addition to favoring early recurrence after cardioversion and the progression of paroxysmal forms to more persistent forms of arrhythmia.^8^Genetic factors can be related to defects in ionic channels and a predisposition for AF. Familial forms of arrhythmia, albeit rare and heterogeneous, are well-described in the literature.^9 , 10^The role of genetics in AF is being studied and represents a promising path in the increasingly modern search for methods of personalized treatment.

Currently, the most widely accepted theories for the initiation and maintenance of arrhythmia are the presence of ectopic foci as triggers and reentry as a maintenance factor. Initial studies already indicated that the topical application of stimulating substances such as aconitine (an alkaloid able to cause bradycardia and hypotension) in the atrium promoted rapid atrial tachycardia, which in turn induced AF.^11^The crucial study for understanding the focal origin of AF was conducted by Haïssaguerre et al.^12^the authors mapped atrial electrical activity in patients with AF and observed early ectopic foci that preceded the occurrence of arrhythmia and mainly originated inside the pulmonary veins ( Figure 2 ).

**Figure 2:** – Triggering foci of atrial fibrillation in various points of the atria (dark spots) predominantly originated in the pulmonary veins. Adapted from Haïssaguerre M, Jaïs P, Shah DC, et al.12 Spontaneous initiation of atrial fibrillation by ectopic beats originating in the pulmonary veins. N Engl J Med. 1998;339(10):659–66.

Whereas focal activity is necessary for the initiation of AF, an atrial substrate favorable for AF maintenance is equally important. Structural, anatomical, and electrophysiological characteristics are essential for the occurrence and maintenance of reentry circuits, which are currently considered fundamental in the maintenance of arrhythmia. Reentry can be anatomical (with obstacles that create slow conduction zones, such as fibrosis) or functional (homogeneous refractoriness resulting from the erratic propagation of the atrial electrical activation wavefront). These conditions increase the probability of multiple simultaneous reentry waves, contributing to the perpetuation of AF.^13^

Autonomic activity also plays an important role in the initiation and maintenance of AF.^14^Vagal activation can alter acetylcholine-activated potassium currents, with consequent reduction of action potential duration; this may stabilize reentry circuits.^15^Moreover, adrenergic activation can cause intracellular calcium accumulation, which could trigger arrhythmia.

Changes in the atrial myocardium structure, particularly fibrosis, separate muscle fibers and interfere in the continuity of electrical impulse conduction, resulting in a reduced conduction speed fundamental for reentry.^16^Fibrosis leads to AF progression, potentially representing a therapeutic target^17^and a predictor of treatment response.^18^Although electrophysiological factors, such as electrical remodeling, and morphological factors, such as fibrosis and atrial dilation (structural remodeling), are considered the main factors involved in AF pathophysiology, increasing evidence has reported that infectious or inflammatory processes can permeate and unite these two situations. A case-control study with 56 870 participants evaluated the association between influenza virus infection, vaccination, and risk of AF. The authors demonstrated that infection increased the risk for developing arrhythmia, while vaccination presented a protective effect in different groups of patients.^19^The presence of inflammatory infiltrate, cellular necrosis, and interstitial fibrosis was higher in patients with AF with no register of structural cardiac disease when compared to patients without arrhythmia.^20^These studies have demonstrated a higher concentration of mediators or markers of inflammatory activity such as interleukin-6 or C-reactive protein (high sensitivity) in patients with AF.^21^

### Risk Factors for Atrial Fibrillation

The high number of AF cases observed in clinical practice is not only justified by the patients’ age; other factors also contribute to this outcome. Since the 1990s, the Framingham study of multivariate analyses has demonstrated that hypertension, diabetes, heart failure, and valvular disease, in addition to age, are independent predictors of this rhythm abnormality.^22^However, various other risk factors have recently been implicated and changes in quality of life indicate a reduction in AF cases, thus becoming a new pillar for excellence in the treatment of AF.^23^

### Obesity and Atrial Fibrillation

Obesity, defined as a body mass index (BMI) of over 30 kg/m^2^, shows clear association with the occurrence of AF. An important meta-analysis including 51 studies and 626 603 individuals demonstrated a 29% increase in the risk of AF for each 5-unit increase in BMI. In addition, risks for postoperative and post-ablation AF considering the same weight increment were also 10% and 13% higher, respectively.^24^Progression of the disease from the paroxysmal to the permanent form is also more significant in obese patients, as reported by a longitudinal cohort study with a 21-year follow-up.^25^Genetics also seems to justify this association. A study with over 50 000 individuals demonstrated that genetic variants associated with high BMI were correlated with the incidence of AF, suggesting a causal relationship between the two conditions.^26^

From this knowledge, many prospective studies have been conducted for demonstrating the impact of weight reduction in AF recurrence.^27 - 32^The LEGACY study (Long-Term Effect of Goal-Directed Weight Management in an Atrial Fibrillation Cohort) included 355 patients followed up for 4 years and divided into 3 groups according to the weight loss at the end of the study. Researchers observed a 6-fold higher probability of being free of rhythm abnormalities in participants who lost (and maintained) more than 10% of body weight when compared to those who lost less than 3% or gained weight in the same period.^28^Another prospective and observational study evaluated 149 patients with BMI values over 27 kg/m^2^who were subjected to AF ablation and to an in-person weight reduction program; these patients presented longer arrhythmia-free survival when compared to the control group.^27^Similar results were observed in a prospective study with 4021 obese patients in sinus rhythm and with no previous history of arrhythmia. Groups underwent to bariatric surgery or to conventional treatment. The weight loss observed in the intervention group was associated with a significant reduction in the risk of AF.^33^

On the other hand, a secondary analysis of the Look AHEAD study (Action for Health in Diabetes), which analyzed patients with diabetes, did not observe a reduction in AF occurrence with the implementation of a weight loss and physical activity program.^34^Another population-based study demonstrated that low lean body mass was also related to the presence of AF.^35^Therefore, the real role of body fat distribution in arrhythmogenesis still requires further clarifications; however, obesity should be recognized as a potentially modifiable risk factor, since a 10% minimum reduction in body weight could decrease the risk of AF in obese and overweight patients.

### Obstructive Sleep Apnea

Obstructive Sleep Apnea (OSA) is characterized by the complete or partial recurrent obstruction of the upper airway, resulting in periods of apnea, oxyhemoglobin desaturation, and frequent nocturnal awakenings. The recognition of this sleep disorder by cardiologists has become fundamental after publications showed an increase in mortality from cardiovascular causes in patients with untreated OSA.^36^Many factors contribute to cardiovascular damage in these patients, and numerous mechanisms may possibly be involved. However, 3 main factors deserve attention: intermittent hypoxia, frequent awakenings, and alterations in intrathoracic pressure. These alterations trigger sympathetic nervous system hyperactivity, endothelial dysfunction, and inflammation.^37 - 40^The sympathetic activation observed in these patients is an important factor that partially justifies the high prevalence of cardiac arrhythmias in this population, including AF. Moreover, OSA can damage left atrial function. Studies with three-dimensional echocardiography demonstrated left atrial dysfunction and remodeling, which were reversed after effective treatment with positive pressure.^41 , 42^

In an epidemiological study, the occurrence of nocturnal cardiac arrhythmias was more frequent in patients with severe OSA, which was defined as an apnea/hypopnea index (AHI) of over 30 events per hour. Atrial fibrillation occurred in 1.65% of cases with severe OSA and in 0.2% of controls (p = 0.03).^43^Another analysis of outpatients followed up for chronic AF in a tertiary hospital and subjected to basal polysomnography discovered that 81.6% presented OSA.^44^OSA and AF are conditions that share risk factors such as age, sex, obesity, hypertension, and heart failure, hence a causal demonstration is challenging in the scientific literature.

In a prospective study^45^with patients referred for electrical cardioversion of AF/atrial flutter, 82% of patients with OSA who received no or inadequate treatment presented recurrence, while this number was 42% in patients who received treatment (p = 0.013). In addition, within the group of patients who did not receive treatment, those who presented a higher drop in oxygen saturation during apnea events had even higher recurrence (p = 0.034). Treatment of OSA reduces the risk of AF recurrence not only in patients subjected to electrical cardioversion, but also in those who go through catheter ablation. In a study with 426 patients subjected to pulmonary vein isolation, 62 patients presented OSA confirmed by polysomnography, of which 32 were continuous positive airway pressure (CPAP) machine users and 30 were untreated. CPAP therapy was associated with a higher AF-free survival rate when compared with patients who did not use the machine (71.9% vs 36.7%; p = 0.01). The authors concluded that CPAP therapy in patients with OSA subjected to percutaneous treatment of AF improved arrhythmia recurrence rates, and in cases of OSA without adequate treatment, electrical isolation had low therapeutic potential.^46^A meta-analysis was then performed for determining the role of OSA in patients with AF subjected to catheter ablation; the study concluded that OSA is associated with a higher risk of AF recurrence after ablation (risk ratio [RR] 1.25, 95% confidence interval [CI] 1.08 to 1.45, p = 0.003).^47^

In conclusion, OSA occurrence is high in patients with AF and current data suggest a dose-response relationship between OSA severity and AF recurrence. Adequate treatment of this sleep abnormality reduces clinical AF recurrence even in patients subjected to catheter ablation. Therefore, adequate investigation and treatment (if necessary) are important measures in the clinical management of these patients.

### Physical Activity and Atrial Fibrillation

Physical inactivity is a public health problem associated with the increase in cardiovascular diseases, heart failure, stroke, cancer, obesity, type 2 diabetes, and hypertension.^48^It thus promotes various risk factors for AF, whereas the literature has recently suggested physical inactivity as an independent risk factor for AF. Five population-based studies have demonstrated a clear relationship between physical inactivity and increased risk for AF.^49 - 53^The CARDIO-FIT study (Cardiorespiratory Fitness on Arrhythmia Recurrence in Obese Individuals With Atrial Fibrillation) evaluated the impact of cardiorespiratory fitness gain in the occurrence of AF in obese and overweight patients.^32^Each peak metabolic equivalent gained during follow-up was associated with a 9% reduction in arrhythmia recurrence, even after correction for weight and risk factors. In a study with patients with permanent AF, 12 weeks of moderate to intense exercise were related to a significant increase in quality of life when compared to controls.^54^These findings were reproducible by other randomized controlled studies and the resulting meta-analysis demonstrated that exercise training improves exercise capacity, quality of life, and left ventricular ejection fraction.^55^

On the other hand, the relationship between physical activity and AF appears to be not linear, but a U-shaped curve; that is, its extremes (whether it be sedentary behavior of strenuous exercise) increase the risk of AF.^56^Notably, the strenuous exercise being referred to here relates to exercises performed in extreme doses that exceed recommendations and correspond to a very small percentage of the population. Interestingly, the effect of intense exercise seems to be influenced by sex. A meta-analysis on the subject demonstrated that vigorous physical activity is associated with a significant increase in risk in men (odds ratio [OR]: 3.30; 95% CI 1.97 to 4.63; p = 0.0002); conversely, intense physical activity was even more significant for a decrease in the risk of AF in women.^57^The mechanisms involved in this difference are still not completely elucidated, but the fact is that moderate physical activity should be encouraged as prevention and treatment, and for improving quality of life in patients with AF.

### Other potential modifiable risk factors

The effects of alcohol in atrial remodeling and in the autonomic nervous system can partially justify the higher AF recurrence observed in individuals who use alcohol.^58^A population-based study with 109 230 healthy participants whose alcohol consumption was quantified through questionnaires demonstrated that, in men, the risk of AF increased along with the quartiles for weekly use of alcohol, suggesting a dose-response association. The same was not verified in women.^59^Even more interestingly, alcohol abstinence has recently been reported to be related to a reduction in the recurrence of arrhythmia in patients with AF. A multicenter, prospective, randomized study performed in Australian hospitals selected patients with an alcohol consumption higher than 10 weekly doses who had paroxysmal or permanent AF and who were in sinus rhythm at baseline evaluation. The group was divided 1:1 between continuing usual alcohol consumption and practicing alcohol abstinence. A total of 140 patients were included; AF recurrence occurred in 53% of patients in the abstinence group, while 73% of patients in the control group presented recurrence. Time to first recurrence was longer in the abstinence group, and the total number of events after a 6-month follow-up was significantly smaller in those who interrupted alcohol use in comparison with controls.^60^

Studies that evaluated the relationship between tobacco use and AF initially presented conflicting results; however, a meta-analysis including 16 prospective studies and 286 217 participants demonstrated a higher prevalence of AF among tobacco users, while habit cessation was associated with risk reduction.^61^Tobacco use also negatively influenced the results of interventional AF treatment.^62^

It is worth noting that the use of high doses of corticosteroids has also been related with an increased risk of AF.^63^To the present moment, no convincing data have related the use of caffeine with an increased risk of AF; some studies suggest a modest protective effect.^64^The same happens with anxiety disorders: In a recent population-based study with 37 402 adults, no relationship was observed between anxiety or depression symptoms and AF.^65^

Figure 3 summarizes the main modifiable risk factors related to quality of life.

**Figure 3:** – Risk factors for atrial fibrillation related to quality of life and their respective guidelines. BMI: body mass index; OSA: obstructive sleep apnea; CPAP: continuous positive airway pressure.

*snoring, excessive daytime sleepiness, fatigue, nonrestorative sleep, memory alterations

### Therapeutic Basis for Atrial Fibrillation

Therapeutic management of AF involves a broad knowledge of the patient’s health state and habits and comprehends 4 main pillars: lifestyle changes and rigorous treatment of risk factors; prevention of thromboembolic events; rate control; and rhythm control^66^( Figure 4 ). We will discuss the therapeutic basis related to long-term treatment.

**Figure 4:** Pillars of the therapeutic management of a patient with atrial fibrillation.

### Lifestyle Change and Rigorous Control of Risk Factors

This pillar aims to reduce the modifiable risk factors associated with quality of life and to rigorously treat cardiovascular comorbidities. Therefore, in addition to controlling body weight, treating tobacco use, tackling sedentary behavior, reducing alcohol use, and optimizing sleep quality, a rigorous control of arterial hypertension, diabetes, and dyslipidemia should also be implemented.

Arterial hypertension is deleterious for patients with AF; not only it constitutes a risk factor for thromboembolic events, but it is also associated with a higher probability of bleeding and recurrence of this arrhythmia. A meta-analysis of AF prevention through the use of renin-angiotensin-aldosterone system inhibitors included 87 048 patients from 23 randomized controlled trials and demonstrated that the use of these drugs reduces the probability of arrhythmia in approximately 33%.^67^

A sub-analysis of the SPRINT study (Systolic Blood Pressure Intervention Trial) evaluated strategies of intensive blood pressure control (systolic blood pressure [SBP] > 120 mmHg) or standard treatment (SBP < 140 mmHg) in AF occurrence. After 5.2 years of follow-up, the risk of AF was 26% lower in the intensive control group when compared to standard control.^68^

Studies demonstrating benefits of arterial pressure control in reducing the risk of AF have been reproducible in the literature, including patients with reduced left ventricle ejection fraction;^69 , 70^however, some contradictory results have also been published.^71 , 72^Other factors may possibly influence primary and secondary AF prevention in patients with hypertension and studies are still necessary for better understanding this relationship.

A meta-analysis involving 7 prospective cohort studies and 4 case-control studies, including 108 703 patients with AF, demonstrated that diabetes is associated with a 34% increase in risk for this type of arrythmia, even after adjusting for confounding factors.^73^The pathophysiological mechanisms of this relationship are still being investigated, but could be multiple, including the impacts of diabetes in the autonomic nervous system observed in diabetic neuropathy. Moreover, hyperglycemia is capable of independently increasing sympathetic tone and reducing parasympathetic tone, which could favor the occurrence of arrhythmia. The atrial electrical and structural remodeling associated with oxidative stress also contributes to AF.^74^However, the relationship between diabetes and AF has become even more important with the report that a rigorous glycemic control was associated with a better control of AF. In an analysis with 12 606 patients, 5-year diabetes treatment was associated with a reduction of approximately 30% in AF cases.^75^

Diabetes can also hinder the progression of patients with AF subjected to catheter ablation. A recent multicenter study including 7 high-volume centers in Europe demonstrated a higher AF recurrence within 1 year in the group of patients with diabetes.^76^Glycemic control also appears to favorably influence the progression of patients subjected to ablation. An observational analysis of patients after ablation demonstrated that the use of pioglitazone was associated with a lower need for a second ablation procedure.^77^

The relationship between dyslipidemia and AF is still under investigation: An observational analysis including 2 large databases (MESA and Framingham) demonstrated that high HDL levels were associated with lower risk of AF, whereas high triglyceride levels were associated with a higher risk. No relationship with LDL was observed.^78^Conversely, a prospective population-based study did not find an association between HDL and triglyceride levels and AF, while low LDL levels were associated with a higher risk of AF. Moreover, the use of hypolipidemic drugs did not influence the occurrence of AF.^79^

Actually, these specific analyses aimed at a single risk factor fail to demonstrate combined actions that are usually employed in clinical practice. For evaluating this effect, 281 consecutive patients who had undergone catheter ablation were selected; they had multiple risk factors and were offered an aggressive program for addressing them. Patients who participated in the program presented significantly higher weight reduction and control of arterial pressure, glycemia, and dyslipidemia. As a consequence, these participants presented higher reductions in AF frequency, duration, and symptoms when compared to the control group (p < 0.001).^80^

### Prevention of Thromboembolic Events

AF is a form of arrhythmia where evaluating eligibility for the prevention of thromboembolic events is mandatory. The use of anticoagulants is superior to treatment with aspirin alone or associated with clopidogrel. It should be indicated for all patients with AF, except when these are classified as very low risk or during the validity of contraindications to the use of this drug class.^81^Left atrial appendage occlusion represents a second alternative for preventing thromboembolic events in patients with restrictions to anticoagulant use.

### Heart Rate Control in Arterial Fibrillation

Heart rate (HR) control is an integral part of the treatment of patients with AF and is normally sufficient for reducing symptoms. The therapeutic target of HR has not yet been established in the literature. The RACE study (Rate Control Efficacy in Permanent Atrial Fibrillation) selected 614 patients with permanent AF who were eligible for rate control; patients were randomized into a lenient strategy (resting HR < 110 bpm) or strict strategy (resting HR < 80 bpm and < 110 bpm during moderate exercise). The objective was to evaluate both strategies regarding a composite outcome including death from cardiovascular causes, hospitalization due to heart failure, stroke, systemic embolism, bleeding, and severe arrhythmias. After a 2-year follow-up, no significant changes were observed between the two approaches, and the frequency of symptoms and adverse events was similar between groups.^82^In a subsequent analysis, the lenient strategy was also not associated with adverse cardiac remodeling.^83^

Drugs used for this purpose include beta blockers, calcium channel blockers (diltiazem, verapamil), digoxin, or a combination thereof.^84^It is worth mentioning that amiodarone can be used in selected cases.

Beta blockers are considered first-line drugs for heart rate control in patients with AF owing to their good tolerability, symptom reduction, and functional improvement. Their therapeutic options, doses, and most common adverse effects are demonstrated in Table 1 . It is worth noting that, in case of therapeutic failure, a combination of drugs can be used. In patients with ventricular dysfunction, beta blockers remain the first-choice drug class due to their benefits in this population, and an association with digoxin can be used when necessary. Calcium channel blockers should not be used in patients with heart failure with reduced ejection fraction due to their negative inotropic effect.^84^Finally, atrioventricular node ablation followed by artificial cardiac stimulation represents a therapeutic option in case of failure of the medication-based approach.

**Table 1:** – Drugs used for heart rate control in patients with atrial fibrillation. Adapted from ESC Scientific Document Group.84 2016 ESC Guidelines for the management of atrial fibrillation developed in collaboration with EACTS. Eur Heart J. 2016;37(38):2893-2962

Drugs most frequently used for heart rate control in patients with atrial fibrillation			
		Dose	Adverse effects
Beta blockers	Metoprolol	100 to 200 mg/day	Lethargy, headache, edema, respiratory symptoms, gastrointestinal alterations, dizziness, atrioventricular block, hypotension
	Nebivolol	2.5 to 10 mg/day	
	Bisoprolol	1.25 to 20 mg/day	
	Carvedilol	3.125 to 50 mg, twice a day	
Calcium channel blockers	Diltiazem	60 mg, three times a day (maximum dose 360 mg/day)	Dizziness, malaise, lethargy, headache, edema, gastrointestinal alterations, atrioventricular block, hypotension
	Verapamil	40 to 120 mg, three times a day (maximum dose 480 mg/day)	
Digoxin		0.0625 to 0.25 mg/day	Gastrointestinal alterations, dizziness, blurred vision, headache, proarrhythmic effects in toxic doses

### Rhythm Control in Patients with Atrial Fibrillation

Acute restoration of sinus rhythm and therapy for maintenance of sinus rhythm are important strategies in the management of patients with AF. Although the maintenance of sinus rhythm appears to be intuitively superior when compared to the rate control strategy , there is no strong scientific literature supporting this claim. The multicenter AFFIRM study randomized patients with AF to these two treatment strategies; they evaluated 4060 patients with a mean age of 69.7 years, 70.8% of which presented arterial hypertension and 38.2%, coronary artery disease. The study reported 310 deaths among patients in the rate control group and 356 among those performing rhythm control after a mean follow-up of 3.5 years (maximum 10 years) (p = 0.08). Moreover, the group subjected to rhythm control presented more adverse effects to medications and a higher number of hospitalizations.^85^A similar result was observed in the RACE study, where the primary outcome (death and cardiovascular morbidity) occurred in 17.2% of patients following the rate control strategy and in 22.6% of those performing rhythm control after a 2.3-year follow-up (p = 0.11).^86^

Although these studies did not present advantages of rhythm control for survival, some aspects are worth mentioning. A sub-analysis of the AFFIRM study using models for determining relationships between survival, baseline clinical variables, and time-dependent variables demonstrated that the presence of sinus rhythm and anticoagulant use were associated with a lower risk of death. On the other hand, the use of antiarrhythmic drugs was associated with higher mortality after adjusting for sinus rhythm. These data suggest that the benefit of sinus rhythm may have been overlooked and alternative methods for maintaining sinus rhythm with less adverse effects could be promising.^87^Another criticism of these results refers to the short follow-up period. In fact, in a population-based analysis with a follow-up period of more than 5 years, mortality was 41.7% in the group subjected to a rhythm control strategy and 46.3% in the rate control group.^88^Therefore, one should consider that the choice between controlling rhythm or rate should be individualized and this is frequently a dynamic process. In a certain moment, the rhythm control strategy may be attractive, but in older patients with less pronounced symptoms, rate control may constitute an alternative.

Acute restoration of sinus rhythm is performed through chemical or electrical cardioversion according to the current protocols. For the subsequent maintenance of sinus rhythm, long-term use of antiarrhythmic drugs, catheter ablation, or the association of strategies are possibilities that should be discussed with the patient. The use of antiarrhythmic drugs for maintaining sinus rhythm is common in the clinical management of patients. Table 2 shows the available drugs used with this objective in Brazil, with their respective doses and adverse effects. It is important to mention that the adverse effects of antiarrhythmic drugs used in the long term are countless, and Table 2 displays the most common or severe ones. In fact, the choice of antiarrhythmic drugs is established more for their safety profiles than for their efficacy. A classic example is amiodarone: Despite presenting a superior rhythm control effect in comparison with other antiarrhythmic drugs, its use is restricted to patients with heart failure due to important toxic effects of its long-term use.^81^Propafenone and sotalol are predominantly used in patients with no structural heart disease; notably, sotalol can cause QT interval prolongation and electrocardiographic monitoring is recommended when employing these medications.

**Table 2:** – Antiarrhythmic drugs used for the maintenance of sinus rhythm

Drugs used for the maintenance of sinus rhythm		
	Dose	Adverse effects
Propafenone	150 to 300 mg, three times a day	Vertigo, heart palpitations, cardiac conduction disorders, bradycardia, tachycardia, anxiety, sleep disorders, headache
Sotalol	80 to 160 mg, twice a day	Bradycardia, dyspnea, chest pain, heart palpitations, syncope, dizziness, diarrhea, nausea, vomiting, fatigue, rash, torsade de pointes
Amiodarone	100 to 200 mg/day	Neutropenia, agranulocytosis, bradycardia, tachycardia, torsade de pointes, hypo and hyperthyroidism, optic neuropathy, neuritis, pancreatitis, elevated transaminase levels, acute liver injury, confusional state, interstitial pneumonitis, bronchospasm, eczema, urticaria, hypotension

Catheter ablation aiming at the electrical isolation of pulmonary veins is an interventional procedure widely used for the prevention of AF recurrence. Overall, catheter ablation is superior to antiarrhythmic drugs for maintaining sinus rhythm;^89^it is currently indicated in symptomatic patients with paroxysmal or persistent AF refractory or intolerant to at least one antiarrhythmic drug, or as first-line treatment of symptomatic paroxysmal AF according to patient preferences. Other individualized indications may also occur. The CABANA study compared catheter ablation and optimized drug therapy in patients with paroxysmal and persistent AF according to the composite outcome of total mortality, stroke, major bleeding, and cardiac arrest. After a follow-up of 5 years, no significant differences were observed between both strategies,^90^but quality of life analyses demonstrated significant clinical improvement and a superior quality of life in patients subjected to ablation.^91^

### Integrated Care of Patients with Atrial Fibrillation

Offering the complex necessary actions for achieving excellence in the care of patients with AF is challenging in clinical practice. The institution of lifestyle changes, rigorous control of risk factors, and promotion of adequate anticoagulation, on top of decisions related to different therapeutic strategies, when centered around a single professional, could produce unsatisfactory results. In this sense, organizing health care services with interprofessional teams when treating patients with AF is fundamental for ensuring the best care. In fact, a randomized study comparing usual care with multidisciplinary care demonstrated a reduction of 35% in relative risk for the composite outcome of hospitalization and mortality.^92^Another important aspect lies on the fact that the complete absence of AF events is often utopic, and treatment should aim to provide improvements in quality of life, promote cardiovascular prevention, and mitigate clinical recurrences.
